# The Royal Alfred Aged Merchant Seamen's Institution

**Published:** 1894-02-03

**Authors:** 


### THE ROYAL ALFRED AGED MERCHANT SEAMEN'S INSTITUTION.

Admiral McClintock writes on behalf of the above institution: "The Royal Alfred Aged Merchant Seamen's Institution endeavours to alleviate the hard lot of as many as possible of our aged, infirm, destitute, and helpless seamen. It maintains about 100 in "The Home" at Belvedere, and it supplies a small pension to about 250 others. Most distressing are the despairing appeals of those whom, for lack of means, the institution is unable to relieve; and most intensely do the committee feel their powerlessness to aid more than a small proportion of those who apply to them. Official records show that out of our 300,000 British merchant seamen, at least 2,300 annually lose their lives by shipwreck; but no records exist of the far greater number disabled by reason of accidents, diseases, and premature old age, brought about by the terrible hardships to which they are exposed by the very nature of their dangerous calling. The charitable public appear to have strangely overlooked the urgent claims of these men, who belong to the most necessary, and perhaps the most deserving class in the community. Will you allow me to plead earnestly for these sufferers—whose best years have been spent at sea—in supplying the needs of our resident population; surely they claim recognition, sympathy, and generous help. The committee of the institution desire to save at least 300 more of them from utter destitution or the workhouse; but if this is to be accomplished, additional subscriptions to the extent of £5,000 are absolutely necessary. Annual subscriptions and donations will be thankfully received by the bankers of the institution, Williams, Deacon and Manchester and Salford Bank, Birchin Lane, E.C.; or by the Secretary, Mr. W. E. Denny, at the office, 58, Fenchurch Street, London, E.C.

